# Process evaluation of a peer-led antenatal breastfeeding class for fathers: perceptions of facilitators and participants

**DOI:** 10.1186/s12884-019-2198-6

**Published:** 2019-01-29

**Authors:** Lesley Kuliukas, Yvonne L. Hauck, Anita Jorgensen, Kelly Kneebone, Sharyn K. Burns, Bruce R. Maycock, Jane A. Scott

**Affiliations:** 1 School of Nursing, Midwifery and Paramedicine, Curtin University, GPO Box U1987, Perth, Western Australia 6845 Australia; 2 King Edward Memorial Hospital, Subiaco, Western Australia Australia; 3 School of Public Health, Curtin University, Perth, Western Australia Australia; 4 Collaboration for Evidence, Research and Impact in Public Health (CERIPH), Curtin University, Perth, Western Australia Australia

**Keywords:** Breastfeeding, Fathers, Antenatal classes, Peer-support, Volunteers, Motivation, Satisfaction, Mixed-methods

## Abstract

**Background:**

The Parent Infant Feeding Initiative (PIFI) was a factorial, randomised controlled trial that aimed to prolong exclusive breastfeeding by targeting expecting fathers. One of the intervention strategies evaluated was a father-focused breastfeeding class facilitated by a male peer facilitator. The aim of this mixed-methods descriptive study was to 1) evaluate the feedback provided from participants of the class and 2) explore the motivations and experiences of volunteer male peer facilitators trained to deliver the class.

**Methods:**

Father-focused breastfeeding antenatal (FFAB) classes were conducted in six Western Australian hospitals between August 2015 and December 2016. Following each peer facilitated FFAB class, expecting father participants completed an evaluation form to assess their satisfaction with the format, facilitation and content, in addition to whether their expectations and confidence to manage breastfeeding problems had changed. Feedback to open-ended questions was analysed using content analysis to identify learnings and suggestions for improvements. At the completion of PIFI, individual telephone interviews were undertaken with 14 peer facilitators to gain insight into their motivations for volunteering and experiences of conducting the classes. Transcripts from interviews were analysed using Braun and Clarke’s six phases for thematic analysis.

**Results:**

Participant evaluation forms were completed by 678 of the 697 father participants (98%). Overall satisfaction with class format, facilitation and content was high with 90% or more of fathers either strongly agreeing or agreeing with each positively-phrased evaluation item. Class participants enjoyed interacting with other fathers, appreciated validation of their role, were not always aware of the importance of breastfeeding or potential difficulties, valued the anticipatory guidance around what to expect in the early weeks of parenting and appreciated learning practical breastfeeding support strategies. Peer facilitators indicated they felt well prepared and supported to conduct FFAB classes. Analysis of interview transcripts revealed common experiences of the peer facilitators incorporating four themes: ‘Highlights of being a facilitator’, ‘Challenges’, ‘Mourning the project completion’ and ‘Satisfaction with training and support’.

**Conclusion:**

Father-focused breastfeeding classes supported by volunteer male peer facilitators are a feasible and acceptable way of engaging fathers as breastfeeding supporters.

**Trial registration:**

ACTRN12614000605695. Registered 6 June 2014.

## Background

There is convincing empirical evidence that the positive attitudes and support of her partner exert a strong influence on a woman’s decision to breastfeed, and are critical to the successful initiation and duration of breastfeeding [1–4]. The importance of getting the ‘right help at the right time’ has been acknowledged by Australian breastfeeding mothers [5] and during times of need, individuals turn to social relationships for social support when they are unable to access the health-care system or when they find it deficient [6, 7]. Given the ready availability of fathers to support their breastfeeding partners, failing to recognize and capitalize on the father’s role as a key support person represents a lost opportunity. International researchers have already established that women who have strong social support from their partner are more likely to initiate and continue breastfeeding [8].

A qualitative study of Swedish fathers’ experiences of their prenatal preparation identified their desire to acquire knowledge and form realistic expectations around parenthood that included developing strategies to adjust to being a father and being supported as a new father [9]. However, a meta-synthesis of 23 qualitative studies involving fathers’ encounters during childbirth suggests that although fathers feel they are a partner and parent, experiences within maternity care contribute to their feeling excluded and fearful [10]. These findings are replicated in studies specific to the perceptions and experiences of fathers with regards to their role as breastfeeding supporters [11].

Research suggests that fathers are generally supportive of breastfeeding but that they lack knowledge of the benefits of breastfeeding, the risks associated with formula feeding, and the knowledge and skills to support their partner to breastfeed [8]. In particular, fathers are generally unaware of how challenging breastfeeding can be and want more information and preparation with a focus on problem solving, so they are better able to support their partner [9]. However, while they are encouraged to attend antenatal classes with their partners, these classes are generally directed at the mother, focused on the birthing process with limited discussion of breastfeeding, and in general do not directly acknowledge or address the support role that the father can provide [12]. Resolving this issue is paramount given that fathers’ participation across the childbearing continuum is associated with long-term involvement and improved child development outcomes [13].

The perinatal period provides an opportunity for connecting with fathers at a time when they are experiencing change, highly motivated and looking for support [14]. However, father engagement in parenting programs is often low with a commonly cited reason being the lack of a male presence in these programs [15]. Maternity services are staffed predominantly by females and entering this environment can be an intimidating experience for fathers [15]. Father-led peer support programs provide a means of addressing this barrier to active engagement. The underlying rationale for father-led peer support breastfeeding programs has been succinctly expressed by Stremler and Lovera: “peer dads become role models and can share information and suggestions in a non-authoritative manner that allows fathers to gain information and confidence in their ability to make decisions and care for their families” (p418) [16]. Fathers are likely to feel less intimidated in sharing their concerns and asking questions of a peer-father compared to a female health professional [16].

Relatively little attention has been paid to the effectiveness of breastfeeding peer support programs which target the father [17–20]. Furthermore, while peer support is acknowledged as an effective strategy to supplement the services of health professionals, the experiences of the peer support personnel are generally not explored. In particular, the experiences of the male facilitators in providing support to expectant fathers have not been investigated. The aim of this study therefore, was to: 1) evaluate the feedback provided from participants of a father-focused antenatal breastfeeding class (FFAB class); and 2) explore the motivations and experiences of the volunteer male peer facilitators who were trained to deliver the FFAB class.

## Method

This mixed-methods descriptive study was part of the larger Parent Infant Feeding Initiative (PIFI) which had the primary goal of prolonging exclusive breastfeeding. PIFI was a factorial randomized controlled trial [21], which evaluated the effectiveness of two father-focused breastfeeding interventions both singly and in combination [22]. One intervention included a single FFAB class facilitated by a volunteer peer facilitator. The other intervention was a smartphone breastfeeding app specifically designed for fathers [23].

### Participants and setting

PIFI participants were expecting couples, attending antenatal classes conducted in three public and three private hospitals in Perth, Western Australia between August 2015 and December 2016. Expectant women are encouraged to attend antenatal classes in their third trimester usually between 28 and 36 weeks gestation. The median gestational age of mothers at recruitment into PIFI was 33 weeks (IQR 31–34 weeks). Inclusion criteria for PIFI were: ownership of a compatible smartphone; internet access; resident of Western Australia; intention of both parents to participate in the rearing of their child; and sufficient English language skills to engage with the intervention.

### Intervention

The format and content of the FFAB class was based on a ‘dads only breastfeeding class’ trialled in the Fathers Infant Feeding Initiative [24], a pilot intervention shaped by the Health Belief Model [25] which identified fathers’ needs and expectations. Social Cognitive Theory [26] informed the overarching PIFI trial and facilitated understanding of the potential interaction between overestimation of new parents’ capacity to cope and underestimation of potential problems [24]. The primary purpose of the FFAB class was to discuss ways that fathers can encourage and support their partners with breastfeeding. The FFAB class explored what it means to be a new dad, the importance of breastfeeding, barriers and facilitators of breastfeeding, and anticipatory problem solving strategies for addressing common breastfeeding problems. It included a series of activities exploring issues identified in the literature [9, 11, 27, 28] and through the Fathers Infant Feeding Initiative process evaluation [24], as being important to meet the needs of new fathers with regards to preparing for fatherhood and supporting breastfeeding. Table 1 presents an overview of the content using the language and style used during the group activities.

**Table 1:** Overview of the Father-focussed Antenatal Breastfeeding Class Activities

Activity	Topic	Duration (min)
1	Introductions and ‘house rules’	5
2	Why breastfeed? The importance of breastfeeding	10
	Key messages: • Breastmilk is more than just food. Breastfeeding is nutritionally superior, cheaper, safer and more convenient than bottle feeding. Breastfeeding has health benefits for baby and mum.	
	• Consider every breastfeed is a success and that any breastfeeding is better than no breastfeeding	
	• Babies’ stomachs are small and they have to feed regularly day and night to consume enough ‘food’. Breastfeeding is time consuming.	
3	Breastfeeding problems/barriers	5
	Key messages • 4 out of 5 women have some problems with breastfeeding in the first few weeks. However, most of these resolve once mum and baby get the hang of it. • Help is available.	
4	Role and expectations of being a father	10
	Key messages: • It is important to consider fatherhood now.	
	• You are NOT expected to be a super hero. Think about some practical ways you can help and provide support to your partner.	
	• Remember you are your partner’s greatest support and first line of defence.	
5	Lifestyle changes and adjustments	2
	• The five ‘S’ – sleep, sex, socialising, stress and self	
6	Planning for success (coping skills and problem solving)	10
	Key Message • Feeding not the only way for a father to bond with baby	
7	Summing up/Evaluation	5

The FFAB class was conducted as part of the regular antenatal class, at the time in the program when the breastfeeding content was typically presented to couples. Those fathers participating in PIFI left the hospital-delivered antenatal class and participated in a one-off ‘dads only’, peer-facilitated class. In the case when the majority of fathers in an antenatal class were PIFI participants, and at the request of the antenatal educators, PIFI non-participants were included in the FFAB class. Classes ran for 45 to 50 min and were led by a trained peer facilitator. The content was delivered in a non-didactic format using a set of slides, either as a PowerPoint or a flipchart presentation, as discussion starting points, to illustrate the key messages. The FFAB class was conducted in an interactive and relaxed manner, typically around a table, and encouraged men to share and discuss their beliefs and attitudes towards breastfeeding and their expectations and concerns about being a father.

### Recruitment and training of peer facilitators

Potential peer facilitators were required to be a father of at least one child aged 3 years or younger who was breastfed for a minimum of 3 months. They were recruited via emails sent to Curtin University staff and students enrolled in Health Science or Education courses. All but two facilitators either had a university degree or were current students enrolled in a health related degree such as physiotherapy or nursing. In July 2015, 18 potential facilitators received a total of 4 hours of training over two evenings, 1 week apart, to become FFAB class peer facilitators. The training program was developed and conducted by the PIFI research team which included a midwife (YH), dietitian (JS) and health promotion educators (BM, SB). All had experience in designing health education interventions including breastfeeding interventions, train the trainer programs and training peer volunteers. The purpose of the training was to complement the existing breastfeeding knowledge and experiences of the peer facilitators by providing them with information that addressed the breastfeeding-related concerns of fathers identified in the literature and to enhance their group facilitation skills [11, 24, 28]. In keeping with the philosophy and attributes of peer support [6], facilitators were not trained as paraprofessionals to provide clinical breastfeeding information but were trained to recognize their scope of expertise and to refer questions of a clinical nature to the hospital antenatal educator. Use of the word ‘peer’ in this context refers to the facilitators being fathers and participants being expecting fathers, rather than socio-economic peers.

On the first training evening PIFI project staff explained the rationale behind the PIFI study and the role and responsibility of the peer facilitator. A mock FFAB class in which the trainee facilitators acted as the participants was conducted by one of the researchers (BM) who was also a trainer in the original Fathers Infant Feeding Initiative project. This provided the trainees with observational and participatory learning of the content, delivery and timing of the antenatal session. Following the observational session, the key messages of each activity were reiterated and explained to the trainees by PIFI staff who also provided basic training in how to conduct small group education classes and to facilitate interactive discussions. The trainees each received a Facilitator Manual which described in detail the learning objective, key messages and teaching strategies for each activity. They were provided also with a USB containing a copy of the FFAB class PowerPoint slides.

**Fig. 1:**
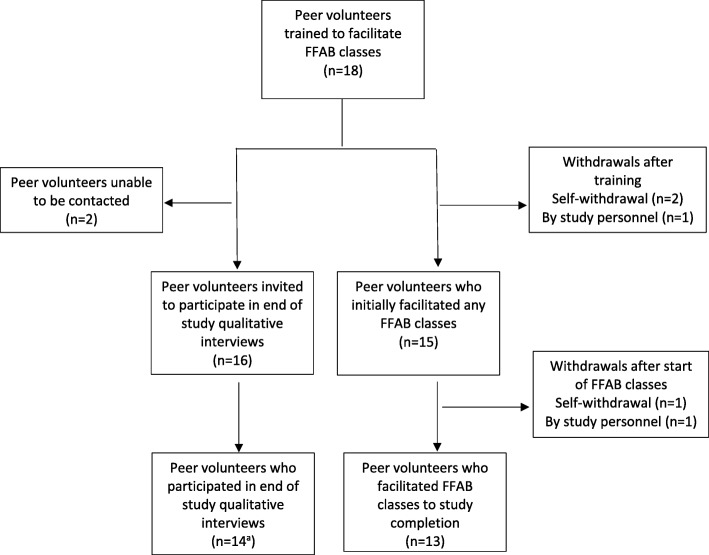
Flow chart of peer volunteers who went on to facilitate father-focussed antenatal breastfeeding (FFAB) classes, and who participated in end of study qualitative interviews. ^a^ Included 12 facilitators who facilitated classes to study completion, 1 who withdrew after delivering 2 classes and 1 who withdrew immediately after training

At the end of the first 2-h training session, each trainee facilitator was assigned one of the FFAB class activities to rehearse and deliver to other trainees in their group the following week. This provided them with the opportunity to deliver one of the class activities and to observe how other trainees delivered the same or a different activity. The trainees received constructive and encouraging feedback on the delivery of their activity from members of the PIFI team and their fellow trainees. All but one of the trainees were assessed by the training team as having sufficient confidence and aptitude at the end of training to deliver the class, and two trainees withdrew (for personal reasons) following training but before delivering any classes. The remaining 15 trainees were assigned a FFAB class at participating hospitals convenient to where they lived or worked (Fig. 1).

Members of the PIFI management team were on-site when the facilitators conducted their first two classes to introduce facilitators to the hospital antenatal educators and assist with setting up audio-visual equipment, but were absent from the room so as not to influence the dynamics of the class. To ensure intervention fidelity, each peer facilitator’s first two classes were audio-recorded, with permission of the participants, and reviewed by members of the PIFI team who provided the peer facilitators with constructive feedback on their delivery, timing and whether they had effectively communicated the key messages for individual activities. It was not logistically feasible to audio-tape and review all subsequent FFAB classes for intervention fidelity, although the results of the participant evaluation were aggregated and reviewed following each FFAB class by a PIFI team member and monitored continuously to ensure that there was no significant drop-off in participant satisfaction. Results of the aggregated anonymous participant feedback were returned to the peer facilitators who were encouraged to reflect on and self-evaluate each FFAB class they delivered throughout the study.

All but two peer facilitators were satisfactorily facilitating classes after two recorded sessions and these two completed a third recorded FFAB class which was reviewed by a member of the PIFI training team. After which, one was sufficiently proficient in facilitating the class and received improved and satisfactory participant feedback, however the second was not assigned any further classes based on the review of the recording and continued unsatisfactory participant feedback. An additional peer facilitator withdrew for personal reasons after delivering 2 FFAB classes. Of the 18 trainees, 13 peer facilitators went on to conduct the remaining FFAB classes between August 2015 and December 2016 (Fig. 1).

### Ongoing peer facilitator support

A closed social-networking site was set up to connect the peer facilitators with each other and the PIFI team, although this was not widely used after the first few months. Peer facilitators were also encouraged to email the PIFI team with any questions they were unable to answer or difficulties experienced in conducting the FFAB class. Problems were addressed promptly and answers to questions and solutions to problems were communicated back to the entire team of peer facilitators via email and the social networking site. For instance, it became apparent in the first few weeks that access to audio-visual facilities in the hospitals was inconsistent and unreliable at all but one of the hospitals, which placed the peer facilitators under unnecessary stress and impacted negatively on the delivery of the class. Consequently, the PowerPoint slides were converted to a set of A3 (297 × 420 mm) posters which were packaged in a briefcase-style table top ‘flipchart’. This proved to be a success with facilitators as they were able to keep the flipchart in their car and did not need additional time for collecting and setting up audio-visual equipment. They also believed that it improved the dynamics of the FFAB class as it allowed them to deliver the session in a conversational style, seated around a table with the participants.

Approximately 6 months after the first FFAB classes were delivered, a further follow-up session was conducted to touch base with the peer facilitators and to obtain their feedback on how the sessions and training materials were working. Facilitators received a $50 gift voucher for either a major variety store or hardware chain for each training session they attended and for each FFAB class they facilitated. They were also reimbursed for any parking expenses that were incurred at some sites. A Christmas Party for peer facilitators and their families was organized by the PIFI management team in 2015 and 2016 to acknowledge their contribution.

### Data collection and analysis

#### Quantitative data

At the end of every FFAB class, father audience participants were invited to complete an evaluation form which assessed their degree of satisfaction with the format, facilitation and content of the FFAB class, and if the class had changed their expectations and confidence to manage breastfeeding problems. The form consisted of nine positively worded statements, and responses were based on a 5-point Likert-scale. For analysis purposes responses were dichotomized into agree (strongly agree/ agree) and neutral/disagree (neither agree nor disagree, disagree, disagree strongly). Chi-square analysis was used to compare the response to items of fathers recruited from private and public hospitals. Knowledge change was not formally evaluated but three open-ended questions invited participants to identify what they had learned during the FFAB class, suggestions for improvements and future sessions and anything else they would like to share with the research team.

#### Qualitative data

Qualitative description is grounded in principles of naturalistic inquiry [29] and is appropriate when first-hand knowledge of participant experiences is required, which in this instance involved facilitation or participation in a FFAB class [30]. Responses offered by fathers to the open-ended questions in the participant evaluation form were analysed using content analysis, an approach commonly used with rich textual data from open ended survey questions [31]. Content analysis provides description through systematic coding and categorising the textual data [32] into common, shared themes representing participants’ experiences [33].

Separate qualitative analysis took place with the male peer facilitators of the FFAB classes. Attempts were made to interview all the volunteers who completed the facilitator training, except for two who were overseas. An email invitation and information letter was sent to the remaining 16 peer facilitators by an independent investigator (LK) who had no prior link to the PIFI study. Individual telephone interviews were undertaken by LK to gain insight into the motivations for volunteering and experiences in conducting the FFAB class across the six metropolitan hospitals. Telephone interviews were audio-recorded with permission and then transcribed for analysis with facilitators being assigned an alias. The interview questions and additional prompts are presented in Table 2. Peer facilitators were asked what the PIFI team could have done to improve their experience in order to determine their satisfaction with the training and support received from the research team. Gathering data from both participant fathers and male peer facilitators allowed for triangulation of data from two data sources thereby providing greater insight into the implementation of the FFAB class [34].

**Table 2:** Interview and prompt questions

We would like to hear about your experiences of being a facilitator for the dads’ sessions in the Parent Infant Feeding Initiative (PIFI) study.	
Prompt questions: 1. What was your motivation in agreeing to be a facilitator for the dads’ sessions? 2. What were the highlights of being a facilitator? 3. What were the challenges of being a facilitator? 4. What kept you engaged to remain a facilitator across the 18 month period? 5. What words of wisdom/advice would you offer to men who may consider a similar role? 6. What could the PIFI team have done to improve your experience? 7. Any final stories/comments you would like to share?	

Transcripts from the peer facilitator interviews were analysed using Braun and Clarke’s [35] recommended six phases for thematic analysis. All transcripts were analysed by LK and other members of the research team analysed a cross-section of transcripts to ensure each data source was reviewed by at least two research team members. Each member presented their preliminary interpretation to the team which continued to meet to negotiate, clarify and refine the final themes, hence maintaining dependability and determining credibility. All disagreements on theme and subtheme descriptions were resolved by referring back to the transcripts. Although data saturation was reached by interview nine, we continued to conduct interviews with all peer facilitators who expressed a desire to share their experiences [36].

## Results

### Participant evaluation

In total, 1426 couples were recruited into the PIFI trial and 671 fathers were randomised to participate in a FFAB class, of whom 600 (89%) attended one of 117 FFAB classes conducted on weeknight evenings or Saturdays between August 2015 and December 2016.

An additional 97 fathers who were non-participants in the PIFI trial also attended one of these classes. On average, each peer facilitator conducted 9 (range 2–22) FFAB classes each and class size ranged from 1 to 11 participants, with 4 to 6 participants being the most frequent size. The average age of PIFI-participant FFAB class participants was 33.9 (SD 5.2) years and 62% had commenced or completed a university level qualification. The majority had been born in Australia (66.1%) or the United Kingdom (12.5%). The characteristics of non-PIFI participants are unknown.

Participant evaluation forms were completed by 678 of the 697 FFAB class participants (98%). Overall satisfaction with class format, facilitation and content was high with 90% or greater either strongly agreeing or agreeing with each of the evaluation items. There was no significant difference in the level of satisfaction between fathers attending a FFAB class at either a private or public hospital (Table 3). Given the overall high level of satisfaction with the FFAB classes, and differences in the group dynamics of each class related to number of participants, no assessment was undertaken to examine facilitator-level differences in participant satisfaction or experiences.

**Table 3:** Proportion of father participants endorsing agreement with evaluation items for FFAB class

Item	Overall % (*n* = 678)	Private % (*n* = 459)	Public % (*n* = 217)	Chi-Square *p*-value
1. I found the session interesting and engaging.	96.8	97.2	96.8	0.778
2. The presenter knew what he was talking about.	97.3	97.4	97.7	0.803
3. The information was delivered at the right pace.	94.2	94.1	95.8	0.355
4. The session environment allowed open discussion.	97.5	98.3	96.8	0.264
5. The information and discussion was relevant.	98.5	99.3	98.1	0.219
6. I have a better idea of what to expect in the early days.	93.2	94.3	92.2	0.294
7. I feel more confident that I will be able to manage problems.	87.8	86.5	90.8	0.106
8. The session helped with my expectations of becoming a father.	90.3	90.8	89.9	0.702
9. I feel better informed about breastfeeding than I was before.	94.4	95.0	94.0	0.596

Major themes and illustrative quotes that emerged from the qualitative analysis of open-ended question are presented in Table 4 and revealed fathers enjoyed interacting with other fathers, appreciated having their role in breastfeeding validated, were not fully aware of the importance of breastfeeding and the potential difficulties that can be encountered, valued hearing about what to expect in the first 4 weeks after their baby arrived and appreciated learning practical ways to support their partner. Suggested improvements included longer sessions and a practical workshop/demonstration element.

**Table 4:** Participant evaluation: analysis of open ended questions

Themes and subthemes	Supporting quotes
Q1 What was the most interesting part of the session for you?	
Session Elements	
Father-Father Interaction	*The opportunity to interact with other dads-to-be*
Learning from a Father	*Learning tips and advice first hand from a dad…*
Validation of Father’s Role	*How the father can make a difference*
Father-focused	*Good to finally have a dad relevant session*
Session Content	
Importance of Breastfeeding	*To hear the benefits of breastfeeding for bub and mum*
Expectations & Explanations	*What to expect in the first few weeks and how to support my wife*
Breastfeeding Difficulties	*Learning that breastfeeding can be difficult and you have to learn how to do it*
Partner Support & Solutions	*I was able to gain a greater understanding of the benefits of breastfeeding and how I can support my wife to enable her to do so successfully*
Bonding	*How to bond with baby, how to be a good dad*
Q2 Please suggest any changes/improvements for future sessions?	
Q3 Is there anything else you would like to tell us?	
Satisfaction	
General Satisfaction	*Great information and well delivered*
Suggestions for Improvement	
More Time, More Interaction	*Bit more time for discussion*
More Information	*More content*
More ‘How To’	*More practical types of support a father can give a mother trying to breastfeed*
More on Expressing	*Storage of breastmilk*

### Peer facilitator experiences

Fourteen of the available 16 volunteers trained to facilitate the FFAB class were interviewed. This included one trainee who withdrew before facilitating any classes and another who withdrew after delivering only a few classes. Analysis of the transcripts revealed common experiences with four themes emerging from the peer facilitators’ stories: Highlights of being a facilitator (with subthemes Making connections and Making a difference), Challenges (with subthemes Giving precious time and Managing sessions that don’t go as planned), Mourning the project completion and final theme: Satisfaction with Training and Support. Quotes are offered in italics to support findings using pseudonyms to ensure facilitator confidentiality.

### Theme 1: Highlights of being a facilitator

The first theme reveals aspects of the facilitator role that were cherished and regarded as particularly memorable. Being in the position of providing support was acknowledged as a highlight by the facilitators: *Being able to have access to that group of dads was a bit of a privilege.... at that prime point in their lives where they’re open to… becoming better dads and changing their lifestyle in some way that’s healthy* (Justin). Two subthemes were captured under this theme: Making connections and Making a difference.

#### Subtheme: Making connections

The facilitators spoke of developing a connection with FFAB class participants: a bond of belonging to the club of being a dad. When Toby was asked about the highlight of being a facilitator he answered: *Getting the interactions with the dads… I suppose it’s kind of building a group of dads that knows that they’re not alone in this situation*. Similarly Daniel found the engagement with fathers satisfying: *That personal interaction was probably one of the highlights*. Oliver spoke about his engaged conversations with participants: *I actually enjoyed the conversations with the guys, that opportunity to have that conversation pretty much was the reason I kept going back.* Equally the facilitators spoke of the benefits of the dads making connections with each other: *How they (the dads) felt it was a really good thing, and that they got a lot out of it… you know that the dads enjoyed it and were able to engage with other dads.*

#### Subtheme: Making a difference

A feeling of satisfaction was noted as facilitators realized they were making a difference and had something tangible to offer these new dads. Justin was approached in the community by a father who indicated: *You were the facilitator at my PIFI group and it was really beneficial and thanks for that … So I thought, I could see that it was having an effect*. Oliver’s self-encouragement was also derived from a belief that positive change was being created: *You know that breastfeeding is a good… there’s nothing better than being able to share that… to make sure that these babies get that advantage in life.*

A sense of altruism and a passion for breastfeeding contributed to facilitators feeling that they would not only be helping the individual dads in their session but also the community, as suggested by Oliver: *it’s just a bit of community service to be honest … it’s a good deed.* At the same time Brian acknowledged the importance of contributing to research: *We do believe in research, so this was an opportunity to volunteer time for something that was research based and will help families. So I think it was the social conscience part of it that we would like to fit into our life.*

Being able to share life experiences of how they assisted their breastfeeding partners contributed to a sense of worth and value. Alex felt he impacted participants’ confidence: *Speaking to people may have helped … not just breastfeeding but becoming a dad and perhaps giving them a little bit more self-confidence.* Giving a sense of reassurance was noted by many facilitators who suggested: *I was keen to help other fathers, new dads, come to grips with what they were about to embark on … dispelling a lot of the myth around it and just letting them know that it’s not that bad, it’s not that scary* (Brad)*.*

### Theme 2: Challenges

The second theme ‘challenges’ acknowledged that facilitating a FFAB class didn’t always proceed smoothly and there were difficulties faced from general, technical and personal perspectives which had to be managed particularly during early sessions when facilitators were unfamiliar with the setting of their assigned hospital. To illustrate, Justin described a technical issue he encountered: *Because the equipment, I had to draw from other rooms and rely on other people and that was problematic.* Other personal challenges raised included being a novice at *public speaking* (Daniel) and introducing embarrassing topics: *I found talking about the sex page a bit hard* (Toby). Challenges incorporated two subthemes.

### Subtheme: Giving precious time

Participants spoke of juggling the time involved in facilitating sessions with other aspects of their lives, made particularly challenging as they had their own parenting duties which they sometimes felt were being neglected. Alex voiced his difficulties with time pressures: *The time factor was difficult for me, just with work and Uni* [university] *and family. So that probably just made it a bit difficult for me at times, to just sort of work around.* For one participant, Matthew, the time commitment made it impossible to continue as a facilitator: *Just the reality of my situation and my time just didn’t really make it feasible.* Being away from home was also mentioned by several participants: *It was difficult to be away during dinner time, the witching hour as some people call it... leaving my wife at home to go out and do this* [Brian]. The defined study duration made it acceptable to Justin but he commented that ongoing sustainability could have compromised his commitment: *I don’t know if I could have sustained it for that much longer being away from my family without some sort of remuneration or something.*

#### Subtheme: Managing sessions that don’t go as planned

There were difficulties with some expecting fathers who were either hard to engage or dominated sessions. Creating a positive learning environment in which all were equally engaged proved to be challenging at times. Brian suggested that some dads were probably coerced into being there: *I really knew we had one or two participants who were probably there because their wife told them to and they weren’t really interested*. Damien shared how drawing out the quiet dads was a concern: *Some would stay very quiet and some would not be too interested.* Facing a session that didn’t proceed as planned was described as exhausting but contributed to facilitators reflecting on how they could have responded differently: *By the time I kind of left … I’m so exhausted after that … Things just weren’t going my way and by the end of the session I was just, oh God I wish I could do that one over again* (Alex).

### Theme 3: Mourning the project completion

Many facilitators expressed a palpable sense of bereavement when the sessions ended, with many lamenting the end of the project and a sense of loss. Not only was a personal loss felt but also a loss for the community which was described by Derek: *It’s a shame it can’t continue forever I know, but it would be good for something similar to continue in some vein.* Justin revealed his personal loss by stating his wish to be involved in any future projects: *I’d like to still be involved with this sort of stuff...if there’s any opportunity I’m happy to be contacted.* Two other facilitators added similar thoughts: *I guess I would have liked to have facilitated more sessions* (Toby) and *If I could do that more often then, I would, it’s something I feel strongly about* (Daniel).

### Theme 4: Satisfaction with training and support

Peer facilitator feedback confirmed that they felt well prepared to undertake their role after attending the training sessions implemented by the research team. *I think the training sessions ran really well … like a team environment from those group training sessions which was really good. It got everyone on the same page as well … so the same message is getting across all channels* (Brad). Should facilitators be confronted with an issue or question they were not prepared for, they felt reassured that the research team was available and would offer appropriate assistance, as Damien shared: *whenever I had a question that I couldn’t answer from my group I would always give it to the researchers … they would go and forward some more information to us*.

Support for their peer facilitator role included the research team demonstrating availability, flexibility and understanding of personal issues that need to be considered when rostering them to FFAB class: *they were understanding of my personal needs* (Alex). Attention to their needs acknowledged the value of their role within this project. *I think they were very well accommodating of the team and they did seem to go out of their way to make sure that we were looked after* (Derek). This was further demonstrated when the research team developed options of either flip chart or a PowerPoint presentation in response to issues with audio-visual equipment availability in some hospital settings: *One of the good things was that they changed from doing … a PowerPoint presentation to actually doing a flip, a flip chart* … *being able to rock up and have everything ready to go, I felt a bit more in control* (Justin).

## Discussion

The FFAB class conducted as part of the PIFI evaluated positively with expecting fathers and appeared to address in part the needs and concerns of expecting and new fathers related to breastfeeding support, as identified through prior research [9, 11, 24, 27, 28]. Fathers who completed the participant evaluation appreciated having an antenatal class which acknowledged and focused on their role as a breastfeeding supporter and allowed them to gain valuable insights from experienced male peer facilitators. Similar results were found with American fathers involved in a father-to-father breastfeeding support pilot program who shared that information received through the program was ‘very important’ and enhanced their knowledge and ability to support their breastfeeding partner [16]. The agencies in the United States (US) that employed peer support fathers also reported an increase in breastfeeding initiation [16]. Another US trial of an educational 2 hour intervention class targeting expecting fathers to enhance their ability to advocate for breastfeeding and support their partner also found a significantly higher difference in breastfeeding initiation in the intervention compared to a control group [17]. These US studies focused upon fathers’ influence on the decision to initiate breastfeeding, however, the influence on breastfeeding duration was either not reported [16] or not significantly different between groups of fathers [17]. The impact of the FFAB classes on the breastfeeding outcomes of PIFI participants are not reported in this process evaluation but will be reported in a separate paper reporting the PIFI outcome evaluation.

Australian fathers in this study appreciated information provided during the FFAB class around the importance of breastfeeding, expectations, potential difficulties, bonding with their child and how to support their breastfeeding partner. In fact, they wanted more information on practical types of support to enhance their preparation in assisting their partner to breastfeed, reinforcing endorsement of this role as a new parent. These findings align with Canadian fathers who confirmed that their role during breastfeeding involved multiple components including participating in decision-making, being responsible for family functioning and providing emotional support [37]. As such, men are receptive to contributing to father inclusive strategies that recognize co-parenting, as demonstrated in a Canadian eHealth breastfeeding resource developed from maternal and paternal feedback that improved breastfeeding self-efficacy, knowledge and infant feeding attitudes of both parents [38].

While peer support is acknowledged as an effective strategy to supplement the services of health professionals [39, 40], the actual experiences of peer facilitators have received less attention. This knowledge is essential to inform sustainability of programs and what is required to recruit and sustain staff who volunteer as peer supporters. Findings from the peer facilitator interviews revealed how they felt well prepared to conduct the FFAB class and how their experiences offered both ‘highlights’ and ‘challenges’. The peer facilitators were recruited through their affiliation with a university as either staff, partners of a staff member or students, and as such represent fathers with a high level of education. This was anticipated, as a known demographic factor related to those who are more likely to be volunteers is higher education [41].

The importance of examining the motivational aspect of volunteering has been recommended [42] and a systemic quality of life (SQOL) theory has been proposed as a new approach to assess volunteering motivation [41]. This approach can clarify the influence of altruism compared to egoism in volunteering which can offer insight into recruitment and retention. The subtheme ‘making a difference’ under the theme of ‘highlights of being a facilitator’ represent qualities such as altruism, empathy, diligence, charity, and benevolence recognized as important for volunteering and further developed during the volunteer process [42].

The Western Australia peer facilitators in this study not only volunteered but, with the exception of three who withdrew for personal or family issues, also demonstrated substantial longevity and sustained commitment to conducting FFAB classes over the 18 months of implementation of the PIFI trial. The themes and subthemes derived from the peer facilitators’ experiences align with the top four ranked SQOL modes renowned as important motivators for volunteering which include: strengthening feelings of belonging to a community; enabling the development of friendships; allowing for the expression of personality and allowing for the expression of beliefs [41]. The ‘highlights’ theme shared by peer facilitators in this study reflect the value of volunteer involvement reinforced in the Canadian code [43] and Australian standard [44]. “Volunteering is about building relationships and connects people to the causes they care about” (p5) [43]. It is personal, promotes belonging and wellbeing.

Standard ten of the Canadian code acknowledges the importance of organisational support and supervision around the volunteer role that provides opportunity for two way feedback [43]. During the training process and early stages of conducting the FFAB classes the peer facilitators were offered constructive and supportive feedback. Additionally, peer facilitators were able to give feedback to the research team that was responded to in a timely manner. For example there were challenges with equipment in some hospitals which led to providing an acceptable and alternate method of presenting information. Support provided in the form of availability, flexibility and prompt responsiveness to issues encountered by the peer facilitators was acknowledged as a valuable component of their volunteering experience. This crucial aspect of support by management aligns with the experiences shared by Canadian health science students during their community-based clinic volunteering that was acknowledged as central and contributed to their level of satisfaction [45]. An additional accidental finding regarding the move from PowerPoint to flip-chart presentation was that the peer facilitators also believed that it improved the dynamics of the FFAB class as it allowed them to deliver the session in a small group style that promoted conversation and discussion. When it works well, small group discussion has been found to allow participants to negotiate meaning, engage more deeply in the subject matter and establish closer contact with facilitators than more formal methods permit [46].

The experiences of peer facilitators conducting FFAB classes within this breastfeeding intervention targeting expecting fathers contributes to a limited body of knowledge in this area. While not previously explored in the context of breastfeeding, the experiences of peer supporters or volunteers have been explored within other contexts revealing rewards and challenges unique to their situation. A phenomenological study with nine Norwegian volunteers working in palliative care shared how their experience was regarded as positive, meaningful and a privilege [47]. Although these evaluations provide valuable information, it is difficult to draw direct parallels to our study due to the nature of the volunteer input in these differing contexts.

The final theme from the analysis of the PIFI peer facilitators’ experiences, ‘mourning the project completion’ captures how these fathers enjoyed and valued their experience as a volunteer in the PIFI study. Key elements aiding the experience; support and communication, appropriate training for volunteers, including written procedures as provided in a Facilitator Manual and being recognized by the PIFI team through social events were also identified in a systematic review on encouragement and support of volunteering from New Zealand [48].

### Limitations

This study was conducted in one city in one Australian state and findings are specific to the father-focused breastfeeding class implemented and assessed antenatally during the PIFI trial. Generalisability cannot be assumed across different international or culturally unique contexts. However, we anticipate that readers may find the content, class activities and training process for the peer facilitators useful to inform development of a father-focused class that is tailored to their particular context. Interview questions to facilitators were focused upon their overall experiences; a direction for further study could be the inclusion of peer facilitators’ perceptions of how participant fathers received the specific information discussed and what aspects they found most useful.

## Conclusion

Breastfeeding education classes for fathers by fathers provided an interactive environment which assisted in understanding the value and importance of breastfeeding. Practical information and advice around providing breastfeeding support for their partners was important to fathers, including learning to deal with potential problems during the early weeks. Fathers also appreciated acknowledgement of their important role in the ongoing success of breastfeeding. Peer father facilitators valued being well prepared by program coordinators to lead the classes and for the ongoing support throughout their role in conducting the classes. The facilitators acknowledged the negative aspects of providing this service, such as the burden of ongoing commitment within their busy lives, however, felt these were outweighed by the positive aspects such as the sense of giving back to society. This process evaluation revealed that father-focused breastfeeding classes are a feasible and acceptable way of engaging fathers as breastfeeding supporters.

## Abbreviations

FFAB: father-focused antenatal breastfeeding class
PIFI: Parent infant feeding initiative
SQOL: systemic quality of life
